# Health State Assessment of Industrial Equipment Driven by the Fusion of Digital Twin Model and Intelligent Algorithm

**DOI:** 10.1155/2022/7324121

**Published:** 2022-08-31

**Authors:** Shuai Wang, Yabin Wang, Xiaoyu Liu, Jinguo Wang, Zhuo Wang

**Affiliations:** ^1^Army Engineering University of PLA, Shijiazhuang Campus, Department of Equipment Command and Management, Shijiazhuang 050003, China; ^2^State Grid Hebei Electric Power Co., Ltd., Marketing Service Center, Shijiazhuang 050035, China

## Abstract

Equipment health state assessment is of great significance to improve the efficiency of industrial equipment maintenance support and realize accurate support. Using the method driven by the fusion of digital twin model and intelligent algorithm can make the equipment health state assessment more suitable for the “accuracy” requirement of equipment support. Taking the neural network algorithm as an example, this paper studies the method of unit level health state assessment of equipment driven by the fusion of digital twin model and intelligent algorithm. The principle and opportunity of equipment health state assessment based on digital twin model are analyzed, the equipment health state grade is redefined from the data-driven perspective, the selection principles of assessment parameters are established, and the unit level health state assessment model of equipment based on digital twin model and neural network algorithm is established. The proposed method is implemented by programming with Python, and the effectiveness of the method is verified by a case study. It provides support for further research of equipment-level health state assessment and the decision-making of equipment maintenance and provides reference for the study of the combination of digital twin model and other intelligent algorithms for health state assessment.

## 1. Introduction

The introduction should be succinct, with no subheadings. Limited figures may be included only if they are truly introductory and contain no new results.

Industrial equipment is the foundation of production-oriented enterprises, and its health state directly affects the production efficiency of enterprises. In order to improve its core competitiveness, enterprises must try to improve the healthy operation time of equipment and reduce the investment in equipment maintenance support as much as possible, so as to save costs and increase profits. Therefore, production-oriented enterprises urgently need to realize accurate equipment maintenance support.

Facing the characteristics of industrial equipment, such as complex structure and function, various degradation conditions, and various failure modes, studying its performance degradation rules and health state assessment method, and timely assessing the health state of equipment can guide enterprises to make optimal support decisions, which is of great significance for enterprises to carry out accurate support [1–3].

Based on the methods of fuzzy comprehensive assessment [4], combined weighting model [5], neural network model [6], and Bayesian network model [7], research works have studied the equipment health state assessment from the qualitative perspective or probability of failure perspective. However, most of the existing research results on the assessment of equipment health state give the level of failure probability, which cannot solve the problem of when the equipment fails and when to carry out maintenance to achieve the best efficiency. The assessment is vague and cannot meet the requirements of accurate equipment support.

The fusion application of digital twin technology and intelligent algorithm provides a way to solve this problem. After continuous exploration, the digital twin modeling technology of industrial equipment has matured. Using the established digital twin model for data analysis and algorithm implementation has become a new research focus of the practical application of digital twin technology. The combination of digital twin technology and intelligent algorithm can give full play to the advantages of digital twin model in mastering a large amount of equipment historical data, real-time data, and empirical knowledge and give full play to the advantages of intelligent algorithm in finding rules through machine learning, so as to obtain high-precision results. Taking the combination of digital twin technology and neural network algorithm as an example, this paper studies the equipment health state assessment method driven by the fusion of digital twin model and intelligent algorithm.

## 2. Analysis of Equipment Health State Assessment Based on Digital Twin Model

### 2.1. Basic Concepts

Equipment health state assessment is the assessment of the ability of equipment and its components to perform its design functions [8, 9]. Health assessment of equipment can effectively ensure the healthy and safe operation of equipment and provide technical support for equipment maintenance and repair decision-making [10].

Equipment health state assessment based on digital twin model is to assess the health state of equipment by using a large amount of data mastered by digital twin model and intelligent algorithm on the basis of digital twin model.

Equipment is composed of replaceable units that perform different functions. Equipment health state assessment can be divided into equipment level assessment and unit level assessment. The equipment level assessment is to assess the overall health state of the equipment. The unit level assessment is used to assess the health state of the replaceable unit of the equipment. The overall health state of equipment is affected by the health state of each unit. This paper mainly studies the method of unit level health state assessment of equipment in use stage based on digital twin model and neural network algorithm, so as to provide basis for equipment use units to research the equipment level health state assessment and make equipment support decisions.

### 2.2. Principle of Assessment

**Figure 1:**
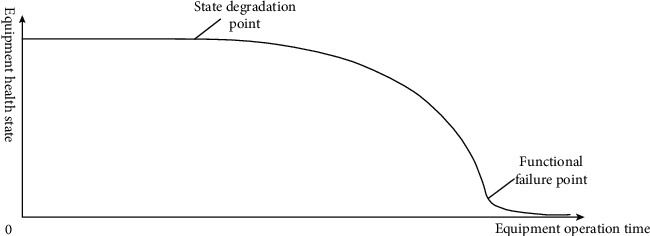
Degradation curve of equipment health state.

With the increase of equipment operation time, the health state of each component of equipment deteriorates according to different rules, and the health degree decreases continuously. The degradation curve of equipment health state is shown in Figure 1 [10].

It can be seen from the figure that the equipment generally does not fail instantaneously but has state degradation after operation for a period of time, and the degradation process is gradually accelerated under the action of various stresses until it is degraded enough to affect the realization of equipment functions, resulting in failure [11]. The degradation curve of equipment health state is an irregular decreasing curve, which cannot be described by analytical method. However, as long as the relevant parameters representing equipment health state are found, the degradation rules of equipment health state can be found.

The purpose of health state assessment is to identify and monitor in a timely manner the relevant parameters that represent the health state of the equipment, to find the best maintenance time between the state degradation point and the functional failure point, to repair the equipment before it is close to failure, and to reduce excessive maintenance and save equipment support cost before ensuring normal operation of the equipment. At the same time, the maintenance equipment is prepared in advance to reduce the waiting time of the equipment [12].

### 2.3. Opportunity of Assessment

Equipment health state assessment based on digital twin model can realize real-time state assessment and master the health state of equipment at any time. In practical work, the combination of centralized assessment and assessment can be adopted at any time. In general, the assessment of the affiliated equipment shall be performed once a day and arranged in the idle time of the equipment at night, so as to avoid affecting the operation of the equipment and the accuracy of the assessment. Under special circumstances, when major changes or emergencies occur to the equipment, it can be assessed at any time. Through the assessment, the health state of the equipment can be mastered in time, and the corresponding countermeasures can be prompted, such as failure early warning, spare parts allocation demand, and equipment maintenance demand.

## 3. Digital Twin Technology and Neural Network Algorithm

Combining digital twin technology with neural network algorithm and giving full play to their respective advantages, we can obtain a high-precision assessment model.

### 3.1. Equipment Support Model Based on Digital Twin

Digital twin is a technology that creates virtual models of physical entities in a digital way. It simulates the behavior of physical entities in the real environment with the help of data and adds or expands new capabilities for physical entities through virtual and real interactive feedback, data fusion analysis, decision iterative optimization, and other means [13]. Based on a large number of advanced technologies such as sensor technology and big data technology, digital twin technology realizes the functions of automatic measurement, automatic recording, active uploading, active analysis, active early warning, auxiliary decision-making, and so forth with high data consistency and reliability; it is favored by research in various fields of production and life, such as engineering manufacturing, aerospace, smart city, smart grid, and system operation and maintenance.

The research of digital twin technology originates from the life cycle management of equipment. It has great matching and many advantages in the application of equipment maintenance support, which can greatly improve the initiative and accuracy of equipment maintenance support. NASA combines the physical system with its equivalent virtual system, studies the failure prediction and elimination method of complex systems based on digital twins, and applies it to the health management of plane, aircraft, launch vehicles, and other flight systems [14]. By combining the ultrahigh fidelity aircraft virtual model with the structural deviation and temperature calculation model affecting flight, the structural science center of the US Air Force Research Laboratory has carried out the life prediction of aircraft structure based on digital twins and summarized the technical advantages of digital twins [15]. Reference [16] introduced the digital twin five-dimensional model into prognostics and health management (PHM) and proposed a method of PHM based on digital twin.

**Figure 2:**
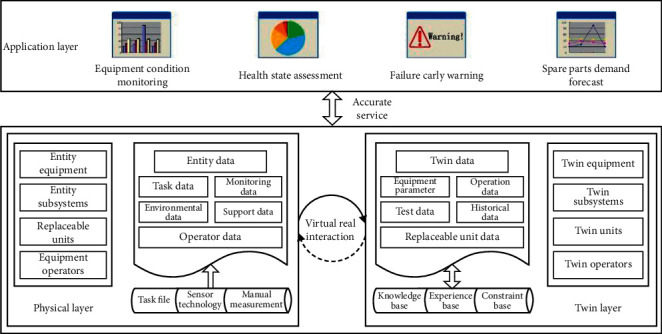
Equipment support model based on digital twin.

At present, the digital twin modeling technology of industrial equipment has matured; [17] has studied and given the equipment support model based on digital twin, as shown in Figure 2.

The equipment support model based on digital twin is divided into four parts: physical layer, twin layer, application layer, and connection layer. In the process of operation, the physical layer transmits the real-time data to the twin layer. The twin layer provides data support to the application layer through data collection and processing and uses the processing results to guide the operation of entities in the physical layer. The application layer uses the data given by the twin layer to provide application services to equipment managers, assist equipment maintenance support decision-making, and act on the physical layer. The connection layer plays the role of internal and external communication of the model, transfers data between various layers within the model, and establishes communication with other relevant digital twin models.

Through the operation of digital twin model, the physical system and the cyber model can be synchronized, making it possible to analyze data online [18, 19]. Using the equipment support model based on digital twin, we can realize the real mapping of virtual equipment model to physical equipment entities, accurately reflect the actual situation of physical equipment, provide support for accurate equipment health state assessment, failure prediction, and spare parts demand prediction, better realize the timely maintenance support of equipment, and reduce the overall support cost [20].

### 3.2. Basic Principle of Neural Network

Artificial neural network system refers to a technical system that uses engineering technology to simulate the structure and function of the human brain neural network. It is a large-scale parallel nonlinear complex network system and is called neural network for short [21]. The neural network has good self-learning ability, nonlinear mapping, and fault tolerance [22] and has been widely used in classification, pattern recognition, prediction, signal processing, expert system, and other fields [23]. As long as there are enough hidden layers and hidden nodes, the neural network can approach any nonlinear mapping relationship, and its learning algorithm belongs to the global approximation method, so it has good generalization ability [24].

**Figure 3:**
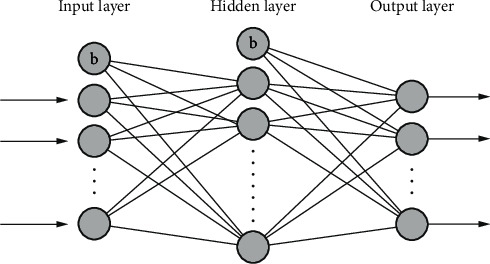
Typical neural network structure.

The typical neural network structure consists of three layers of neurons, namely, input layer, hidden layer, and output layer, as shown in Figure 3. Each layer is composed of several neurons, which are fully connected, and bias nodes (represented by (b) are added in the input layer and hidden layer. Through the continuous correction of the connection weights of each layer, the error convergence is realized, and finally a reliable neural network model is obtained to realize the required functions.

Due to the large difference in the probability of different results, the training dataset of neural network is usually imbalanced, which requires the use of algorithms such as granular computing and random forest to identify the optimal granularity and refine the imbalanced dataset [25, 26].

Programming with Python or other languages can realize the processing of training dataset, as well as the establishment and training of neural network model, and the neural network model that meets the training requirements can be used to realize the functions of data fitting, classification, clustering, and so on.

## 4. Equipment Health State Grade under Data Drive

The health state of equipment is described by health state grade. At present, the research on health state grade is mostly described by qualitative description or failure probability, which cannot adapt to the data-driven health state assessment method. To adapt to the data-driven health state assessment and provide support for the assessment, it is necessary to redefine the health state grade from the data-driven perspective.

**Figure 4:**
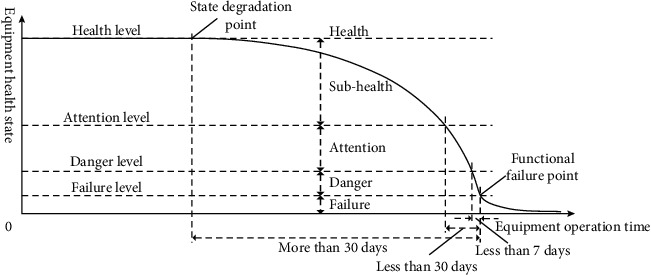
Equipment health state grade.

According to the degradation curve of equipment health state and the support cycle of equipment maintenance equipment, the health state of equipment replaceable units is divided into five grades: health, subhealth, attention, danger, and failure, which are defined from the perspective of digital drive, as shown in Figure 4 and Table 1.

*Health*. The equipment is healthy and has good performance, and all indicators are in good condition, which is suitable for long-term operation. For such equipment, only daily maintenance is required.

*Subhealth*. The equipment performance degrades to a certain extent, but it does not affect the normal operation of the equipment. The failure is almost impossible to occur in the next 30 days. For such equipment, while doing well in daily maintenance, it is necessary to strengthen condition monitoring.

*Attention*. The equipment performance degradation is serious and can be detected obviously. Failure may occur in the next 30 days. For such equipment, it is necessary to strengthen condition monitoring, prepare for repair, and query the inventory of spare parts. If the inventory is insufficient, spare parts shall be allocated in time.

*Danger*. The equipment performance degradation is very serious, which has affected the operation quality of equipment. Failure may occur in the next 7 days. For such equipment, it needs to be repaired immediately during the scheduled downtime.

*Failure*. The equipment failure has occurred and the function of the equipment has been affected. Such equipment can only be shut down for repair.

## 5. Assessment Method of Equipment Health State

**Figure 5:**
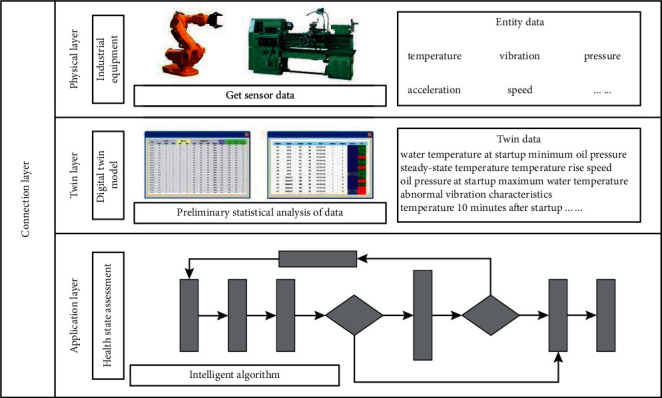
Schematic diagram of the data flow.

In the process of equipment health state assessment driven by the fusion of digital twin model and intelligent algorithm, the physical layer of digital twin model is responsible for connecting with the physical equipment through various sensors to obtain the most real original data and provide the data to the twin layer. The twin layer is responsible for preliminary statistical analysis of the obtained data, obtaining the analyzed twin data and transmitting it to the application layer for use. The application layer is responsible for using the obtained twin data to complete the task of health state assessment through intelligent algorithm. The information transmission between each layer is uniformly scheduled and transmitted through the connection layer. The data flow diagram of equipment health state assessment driven by the fusion of digital twin model and intelligent algorithm is shown in Figure 5.

A large number of papers have been studied on the establishment of digital twin model, as well as the application of sensor and preliminary statistical analysis of data, which will not be repeated in this paper. This paper mainly studies the process of health state assessment in the application layer of digital twin model, combined with intelligent algorithm.

The basic idea of equipment health state assessment driven by the fusion of digital twin model and intelligent algorithm is as follows:

1. Select assessment parameters.
2. Obtain parameter data from the digital twin model.
3. Assess health state through an intelligent algorithm.
4. Give assessment conclusions and suggestions.

**Figure 6:**
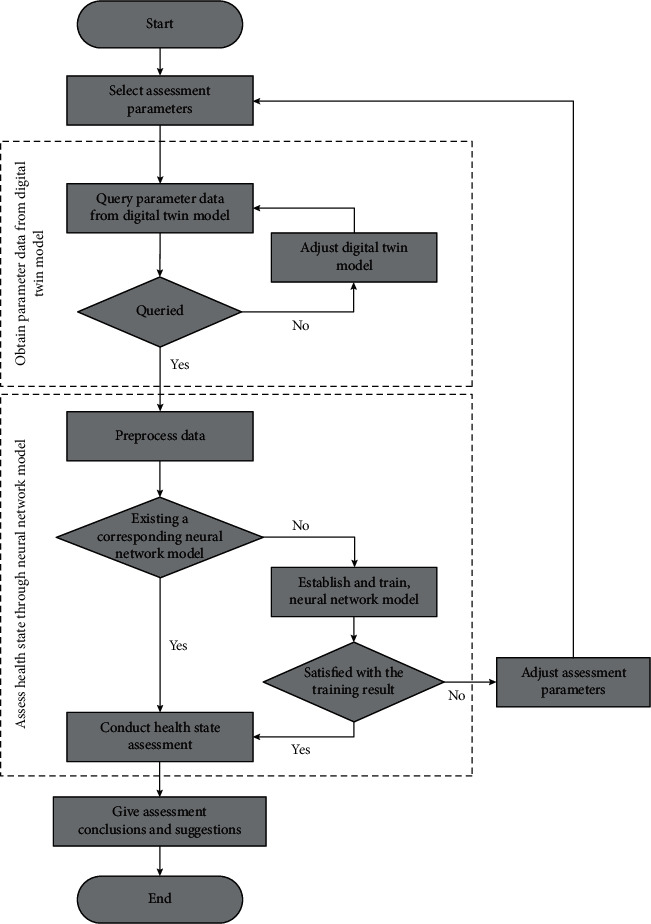
Assessment process.

Taking the neural network algorithm as an example, the specific process of equipment health state assessment driven by the fusion of digital twin model and intelligent algorithm is shown in Figure 6.

### 5.1. Select Assessment Parameters

The health state of equipment can be characterized by a series of state parameters. As long as the parameters are selected comprehensively and reasonably, the health state of equipment can be characterized [27]. The state parameters commonly used in equipment mainly include temperature, vibration, pressure, speed, and acceleration, such as water temperature, oil temperature, oil pressure, amplitude, and frequency. At the same time, the analysis data of relevant parameters after preliminary analysis are also the characterization of the health state of the equipment, such as water temperature at startup, oil pressure at startup, maximum water temperature, minimum oil pressure, temperature rise speed, temperature 10 minutes after startup, steady-state temperature, and abnormal vibration characteristics.

When selecting assessment parameters, attention should be paid to the following aspects:

1. The assessment parameters are not limited to the determinants of equipment health state and can also be related factors of health state, that is, the factors that will change due to the change of health state. As long as it can reflect some or several changes in the health state of equipment, it can be used as the selection object. When selecting assessment parameters, the most closely related state parameters should be selected as much as possible from the perspective of failure inducing mechanism.
2. When selecting assessment parameters, we should take into account the principles of correlation and measurability, should comprehensively analyze and optimize measurable state parameters from the perspective of existing measurement technology, and should not put forward too high requirements for the measurability of parameters. If too many sensors are added, not only will the cost be greatly increased, but also the normal operation of the equipment may be affected. The analysis of equipment state data can be strengthened, and the analysis of relevant data can be used to solve the problem that some data cannot be measured.
3. The selected parameters should not be too many or too few. On the premise of reflecting the changes of equipment health state from the perspective of intelligent assessment, the assessment parameters should be selected as few as possible.

If it is found that the training result is not good enough in the process of training the neural network model, it shows that the correlation between the selected parameters and the equipment health state is not strong; that is, the parameter selection is not reasonable. At this time, it is necessary to reselect the state parameters with strong correlation with the equipment health state as the assessment parameters of the equipment health state.

### 5.2. Query Parameter Data from Digital Twin Model

Using sensor technology, digital twin model can obtain a large amount of state data efficiently, establish the real mapping between the virtual model and the physical entity, and truly reflect the real-time state of equipment. At the same time, the digital twin model has sufficient historical and empirical data, which can be used as the support of data analysis.

Querying parameter data from digital twin model is to query and collect relevant data of assessment parameters in digital twin model, including current data, historical data, and empirical data, as the basis for further analysis. If no relevant data is queried from the digital twin model, the digital twin model needs to be adjusted.

### 5.3. Adjust Digital Twin Model

When establishing the digital twin model, the common parameters of equipment are mainly considered. If some selected parameters are not collected in the digital twin model and it is necessary to collect these parameters for health state assessment, it is necessary to adjust the digital twin model, add corresponding sensors, collect these parameters, and collect relevant historical and empirical data.

### 5.4. Preprocess Data

To meet the needs of neural network algorithm, the input data need to be preprocessed in two aspects: state data normalization and grade data formatting.

(1) State data normalization. The state data of the input data of the neural network model should be normalized according to a certain method to avoid the influence of different data value range on the results. There are many normalization algorithms, and appropriate methods can be selected according to the characteristics of data. Here, a simple method of maximum linear conversion is introduced as an example. The conversion equation is
  (1) $$\begin{array}{c} x\prime =\frac{\left(x-x_{\text{min}}\right)}{\left(x_{\text{max}}-x_{\text{min}}\right)}, \end{array}$$
where *x*' is the value after normalization, *x* is the value before normalization, *x*_min_ is the minimum value of the sample, and *x*_max_ is the maximum value of the sample.
(2) Grade data formatting. The grade data of the input data of the neural network model shall be marked by a one-dimensional array in the format of 0-1. For example, if the health state of the equipment is divided into five grades, the grade data of the five grades shall be represented by a one-dimensional array [1,0,0,0,0], [0,1,0,0,0], [0,0,1,0,0], [0,0,0,1,0], and [0,0,0,0,1], as shown in Table 2.

### 5.5. Establish and Train the Neural Network Model

In the stage of state assessment through the neural network model, if the assessed unit has established and trained the corresponding neural network model in the early stage, it can directly use this model for health state assessment. Otherwise, it should use the data obtained from digital twin model to establish and train the neural network model and assess the training result of the model. The established neural network model should also be retrained once a year.

#### 5.5.1. Design the Model

The number of nodes in the input layer is represented by *n*, which is equal to the number of selected assessment parameters. The more assessment parameters, the more nodes.

The number of nodes in the output layer is represented by *m*, which is the number of required classifications. For example, if the equipment health state is divided into 5 grades, the number of nodes in the output layer is *m* = 5.

The hidden layer is designed as one hidden layer, and the number of nodes is represented by *n*_1_. It is calculated according to (2), and the number of nodes with the best training result is set after many tests within the value range.

(2) $$\begin{array}{c} n_{1}=\sqrt{n+m}+a, \end{array}$$

where *n*_1_ is the number of hidden layer nodes, *m* is the number of output layer nodes, *n* is the number of input layer nodes, and *a* is a constant between 1 and 10.

**Figure 7:**
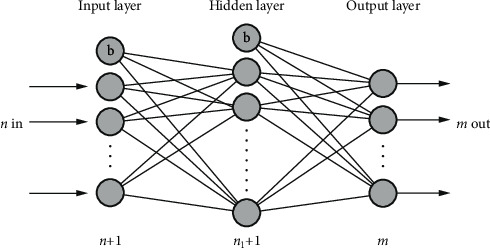
Neural network design.

After adding bias nodes in the input layer and hidden layer, respectively, the neural network design is shown in Figure 7.

#### 5.5.2. Train the Model

Distinguish the input data, so that 75% of the data is used for model training and 25% of the data is used for model testing. Write the neural network algorithm program in Python; we can train the neural network model. By continuously correcting the connection weights of each node in each layer, the error convergence can be realized and a reliable neural network model can be obtained.

#### 5.5.3. Assess the Training Result

After the training of neural network model is completed, the training result of the model can be assessed and the accuracy of the model can be given through the model error and the classification effect of the model on the training data and test data. If the training effect meets the needs of assessment, the model can be used for health state assessment. If the training effect is not good enough, it indicates that the selected assessment parameters are not strongly correlated with the equipment health state. The assessment parameters should be adjusted according to the selection principle of assessment parameters, and the neural network model should be reestablished.

#### 5.5.4. Retrain the Model

The established neural network model should be retrained regularly, and the newly collected actual operation data should be substituted into the model as input data, so as to increase the amount of data and improve the accuracy of the model. Since one year is generally a cycle of equipment operation and support and relatively complete operation and support data can be obtained at the end of the year, the retraining is generally conducted once a year and arranged at the end of each year.

### 5.6. Conduct Health State Assessment

After obtaining the data from the digital twin model and establishing and training the neural network model, we can use the state data and neural network model to assess the health state of equipment. After preprocessing the parameter data reflecting the current state of the equipment according to the same method as the input data and inputting them into the trained neural network model, the current health state grade of the equipment can be obtained, which directly reflects the health state of the equipment.

### 5.7. Give Assessment Conclusions and Suggestions

According to the health state assessment result, combined with the knowledge base and expert system of digital twin model, we can give the health state assessment conclusion, describe the current health state of the equipment, and present equipment maintenance suggestions, so as to help equipment managers better carry out equipment support and give full play to the maximum efficiency of the equipment.

## 6. Case Study

We take the engine fuel system of a certain equipment as an example to assess its health state. The engine fuel system is a replaceable unit of the equipment.

### 6.1. Select Assessment Parameters

According to the characteristics of engine fuel system, the pressure of high-pressure oil pipe is selected as the measurement basis, and six parameters are selected as the assessment parameters of the health state of the engine fuel system, as shown in Table 3.

### 6.2. Obtain and Preprocess Data

3000 groups of historical data can be obtained from the built engine digital twin model as the input data of the neural network model, and the data are preprocessed. Part of the preprocessed data is shown in Table 4.

### 6.3. Establish and Train the Neural Network Model

We can write a program in Python to establish and train the neural network model.

The number of input layer nodes *n* = 6 and the number of output layer nodes *m* = 5 of the neural network model can be determined by 6 state parameters and 5 health state grades. According to (2), the number of hidden layer nodes *n*_1_ can be selected from 4 to 13. Through many experiments, when the number of hidden layer nodes *n*_1_ = 13, the model training effect is the best. Thus, the neural network model can be established.

**Figure 8:**
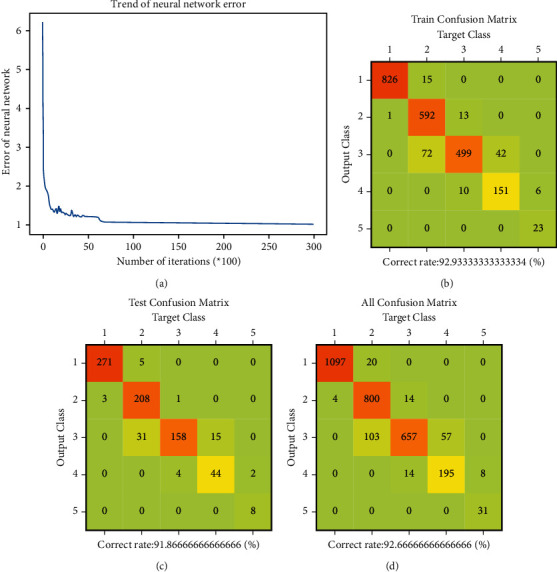
Training result of neural network. (a) Trend of neural network error. (b) Train confusion matrix. (c) Test confusion matrix. (d) All confusion matrix.

The neural network model is trained by using the preprocessed input data. After many times of training, the satisfactory training result is obtained, as shown in Figure 8. It can be seen that the error of the neural network model decreases gradually through training. In the final neural network model, the correct rate of training data is 92.93%, the correct rate of test data is 91.87%, and the correct rate of all input data is 92.67%. The correct rate of the test data is close to that of the training data, and the error results only appear in the adjacent state of the actual results, indicating that the model does not overfit, and its correct rate is high, which can meet the requirements of the assessment model.

### 6.4. Conduct Health State Assessment

After extracting the current state information of the engine fuel system from the digital twin model and preprocessing, the state data can be obtained, as shown in Table 5.

**Figure 9:**
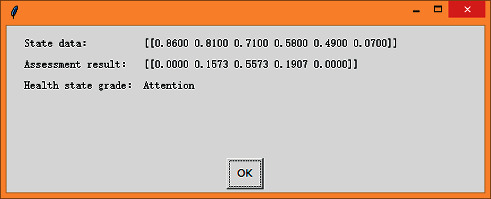
Conduct health state assessment.

After running the neural network model and entering the state data into the model, the assessment result can be obtained, as shown in Figure 9.

It can be seen that the assessment result of the current state is [0.0000, 0.1573, 0.5573, 0.1907, 0.000], suggesting that the engine fuel system is in the “Attention” state at this time. The assessment result is consistent with the actual situation.

### 6.5. Give Assessment Conclusions and Suggestions

Through the assessment, the engine fuel system is in the state of “Attention” at this time. Combined with the grading principle and referring to the knowledge base and experience base in the digital twin model, the assessment conclusion is that the performance degradation of the engine fuel system is serious and failure may occur in the next 30 days. The assessment suggestions are as follows: strengthen condition monitoring, prepare for repair, and allocate spare parts in time.

The above case shows that using the fusion driven method of digital twin model and neural network algorithm can effectively realize the assessment of equipment health state and obtain intuitive and accurate assessment results.

## 7. Discussion

The above method defines a data-driven health state grade and uses the fusion drive of digital twin model and neural network algorithm to assess the health state of equipment and achieves the expected results.

This problem can also be solved by SOTA methods such as fuzzy comprehensive assessment, combined weighting model, and Bayesian network model, but because these methods are mostly assessed from the qualitative perspective or probability perspective, the assessment results are relatively fuzzy, which cannot meet the requirements of equipment support accuracy.

Compared with these methods, this method has the following characteristics:

1. Faster. Using this method, real-time health state assessment can be realized. Because the digital twin model is established in advance and the way to obtain data is established, the data acquisition is faster. Because the training of neural network model is completed in advance and the training results can be used for many times, the assessment process is also faster.
2. More accurate. In this method, the intelligent algorithm is used to make full use of the advantages of big data and find the failure rules suitable for each equipment. It avoids the process of determining the weight through expert scoring in some traditional methods and eliminates the interference of human factors, and the result is more accurate and objective. It meets the needs for accurate support.
3. Better adapt to new requirements. The data-driven state grade is redefined to define the state with data, which meets the needs of big data-driven mode. The complex calculation process of some traditional methods is avoided, and the model can be used many times after training, which reduces the amount of calculation of state assessment and meets the needs of big data processing.
4. More practical. The traditional health state assessment can only be used to count the state of batch equipment and master the overall state of equipment. This method can be used to assess the equipment group, single equipment, and a part of equipment. The assessment results can be used to reliably judge the health state of equipment, guide the support decision-making, and improve the active of equipment support.
5. More automated. From data collection to processing to assessment, the whole process of assessment is automated, which saves labor, improves work efficiency, and greatly reduces the error rate.
6. Portable. For new equipment that lacks a large amount of data support, the parameter data in the assessment model of similar equipment can be transplanted as empirical data to become the initial data of new equipment to bring the initial support data of new equipment closer to reality and provide support for the support decision of new equipment.

## 8. Conclusions

Equipment health state assessment is of great significance to realize equipment accurate support. Taking the neural network algorithm as an example, this paper studies the method of unit level health state assessment of equipment driven by the fusion of digital twin model and intelligent algorithm. The equipment health state grade is redefined from the data-driven perspective, and the unit level health state assessment model of equipment based on digital twin model and neural network algorithm is established. The effectiveness of the method is verified by case study. Support is provided for further research of equipment-level health state assessment and the decision-making of equipment maintenance.

This paper mainly studies the method of health state assessment based on the combination of neural network algorithm and digital twin model. In fact, there are many intelligent algorithms that can be combined with digital twin model for equipment health state assessment. Different equipment or different replaceable units may need to use different algorithms according to their characteristics, but the method of combination with digital twin model is the same. The combination of digital twin model and other algorithms for health state assessment can be further studied in the future.

## Figures and Tables

**Table 1:** Detailed table of equipment health state grade.

Health state grade	Grade identification	State performance	Definition under digital drive	Maintenance measures
Health	1	Good performance, all indicators are in good condition, suitable for long-term operation.	All indicators are in good condition.	Carry out the daily maintenance.

Subhealth	2	The performance degrades to a certain extent, but it does not affect the normal operation of the equipment.	The failure is almost impossible to occur in the next 30 days.	Carry out the daily maintenance and strengthen condition monitoring.

Attention	3	The performance degradation is serious and can be clearly detected.	Failure may occur in the next 30 days.	Strengthen condition monitoring, prepare for repair, and allocate spare parts in time.

Danger	4	The performance degradation is very serious, which has affected the operation quality of equipment.	Failure may occur in the next 7 days.	Repair at the right time.

Failure	5	A failure has occurred and the function of the equipment has been affected.	Failure has occurred.	Shutdown for repair.

**Table 2:** Grade data format.

Health state grade	Grade identification	Grade data format
Health	1	[1,0,0,0,0]
Subhealth	2	[0,1,0,0,0]
Attention	3	[0,0,1,0,0]
Danger	4	[0,0,0,1,0]
Failure	5	[0,0,0,0,1]

**Table 3:** Health state assessment parameters.

Parameter number	Parameter content
Parameter 1	Pressure at the beginning of fuel injection
Parameter 2	Maximum fuel pressure
Parameter 3	Submaximum fuel pressure
Parameter 4	Width of the rising phase of pressure waveform
Parameter 5	Height difference between the highest point and the lowest point of pressure waveform
Parameter 6	Area of pressure waveform in an injection cycle

**Table 4:** Part of the preprocessed data.

Parameter 1	Parameter 2	Parameter 3	Parameter 4	Parameter 5	Parameter 6	Grade identification				
0.28	0.80	0.17	0.51	0.91	0.32	0	1	0	0	0
0.30	0.04	0.25	0.25	0.34	0.05	1	0	0	0	0
0.79	0.33	0.14	0.18	0.69	0.20	1	0	0	0	0
0.53	0.21	0.27	0.85	0.88	0.57	0	0	1	0	0
0.28	0.84	0.24	0.21	0.11	0.90	1	0	0	0	0
0.22	0.61	0.17	0.38	0.52	0.97	0	1	0	0	0
0.80	0.57	0.59	0.60	0.42	0.45	0	0	1	0	0
0.78	0.69	0.17	0.79	0.83	0.32	0	0	1	0	0
0.03	0.54	0.87	0.07	0.04	0.79	0	1	0	0	0
0.76	0.42	0.50	0.49	0.78	0.61	0	1	0	0	0
0.10	0.67	0.06	0.03	0.54	0.48	1	0	0	0	0
0.57	0.62	0.92	0.53	0.48	0.82	0	0	0	1	0
0.96	0.82	0.44	0.16	0.45	0.25	1	0	0	0	0
0.96	0.96	0.10	0.27	0.37	0.42	1	0	0	0	0
0.45	0.15	0.18	0.42	0.66	0.72	1	0	0	0	0
0.90	0.74	0.06	0.11	0.00	0.74	0	1	0	0	0
0.04	0.62	0.58	0.65	0.04	0.51	0	0	1	0	0
0.80	0.16	0.58	0.43	0.88	0.34	0	1	0	0	0
0.83	0.57	0.19	0.51	0.67	0.67	0	0	0	1	0
0.14	0.65	0.65	0.02	0.16	0.10	1	0	0	0	0
0.83	0.83	0.65	0.44	0.21	0.48	0	1	0	0	0
0.08	0.44	0.56	0.56	0.32	0.92	0	1	0	0	0
0.38	0.88	0.16	0.74	0.21	0.05	1	0	0	0	0
0.31	0.75	0.98	0.33	0.18	0.88	0	1	0	0	0
0.50	0.36	0.72	0.12	0.77	0.10	1	0	0	0	0
…	…	…	…	…	…	…	…	…	…	…

**Table 5:** State data after preprocessing.

Parameter 1	Parameter 2	Parameter 3	Parameter 4	Parameter 5	Parameter 6
0.86	0.81	0.71	0.58	0.49	0.07

## Data Availability

The raw data used to support the findings of the study can be obtained from the corresponding author upon request.
